# Fibroblast-driven collagen expansion and altered thymic medullary niches in 22q11.2 deletion syndrome

**DOI:** 10.70962/jhi.20260011

**Published:** 2026-05-04

**Authors:** Viktoria Hennings, Jenny Lingman Framme, Karolina Thörn, Christina Lundqvist, Andri Lemarquis, Solveig Oskarsdottir, Esbjörn Telemo, Åsa Björklund, Olov Ekwall

**Affiliations:** 1 Department of Pediatrics, Institute of Clinical Sciences, The Sahlgrenska Academy, University of Gothenburg, Gothenburg, Sweden; 2 Department of Rheumatology and Inflammation Research, Institute of Medicine, The Sahlgrenska Academy, University of Gothenburg, Gothenburg, Sweden; 3 Department of Pediatrics, Halland Hospital, Halmstad, Sweden; 4 Division of Biomolecular and Cellular Medicine, Department of Laboratory Medicine, Karolinska Institutet, Huddinge, Sweden; 5 Department of Cellular Therapy and Allogeneic Stem Cell Transplantation, Karolinska University Hospital Huddinge, Huddinge, Sweden; 6 City of Hope Los Angeles and National Medical Center, Duarte, CA, USA; 7 Department of Life Science, National Bioinformatics Infrastructure Sweden, Science for Life Laboratory, Chalmers University of Technology, Gothenburg, Sweden

## Abstract

22q11.2 deletion syndrome (22q11DS) is associated with congenital anomalies and variable thymic hypoplasia with T cell lymphopenia and immune dysregulation. However, the spatial organization of human thymic lymphopoiesis and stromal mechanisms contributing to thymic dysfunction in 22q11DS remain incompletely defined. We applied spatial transcriptomic and spatial proteomic analyses on thymic samples from two 22q11DS patients and compared them with healthy controls. Across 22q11DS samples, we observed alterations in the corticomedullary organization and in the frequencies of fibroblasts, B cells, regulatory T cells, and mTEC subsets. These features coincided with a prominent remodeling of the mesenchymal compartment, including increased expression of extracellular matrix programs and collagens, and predicted disruption in mesenchymal–epithelial cell crosstalk. In the medulla, we observed alterations in interferon-associated gene programs within a colocalized niche comprising B cells, antigen-presenting cells, and mTEC subsets. Together, this provides an integrated spatial map of the 22q11DS thymus and nominates stromal remodeling as a candidate driver of impaired central tolerance induction in 22q11DS.

## Teaser

The mechanisms underlying thymic dysfunction in the 22q11.2 deletion syndrome are poorly defined. In this study, Hennings et al. integrated spatial transcriptomic and proteomic analyses of thymus tissue from patients with 22q11.2 deletion syndrome to identify fibroblast-driven collagen expansion and alterations in medullary niches.

## Introduction

**Figure 1.**
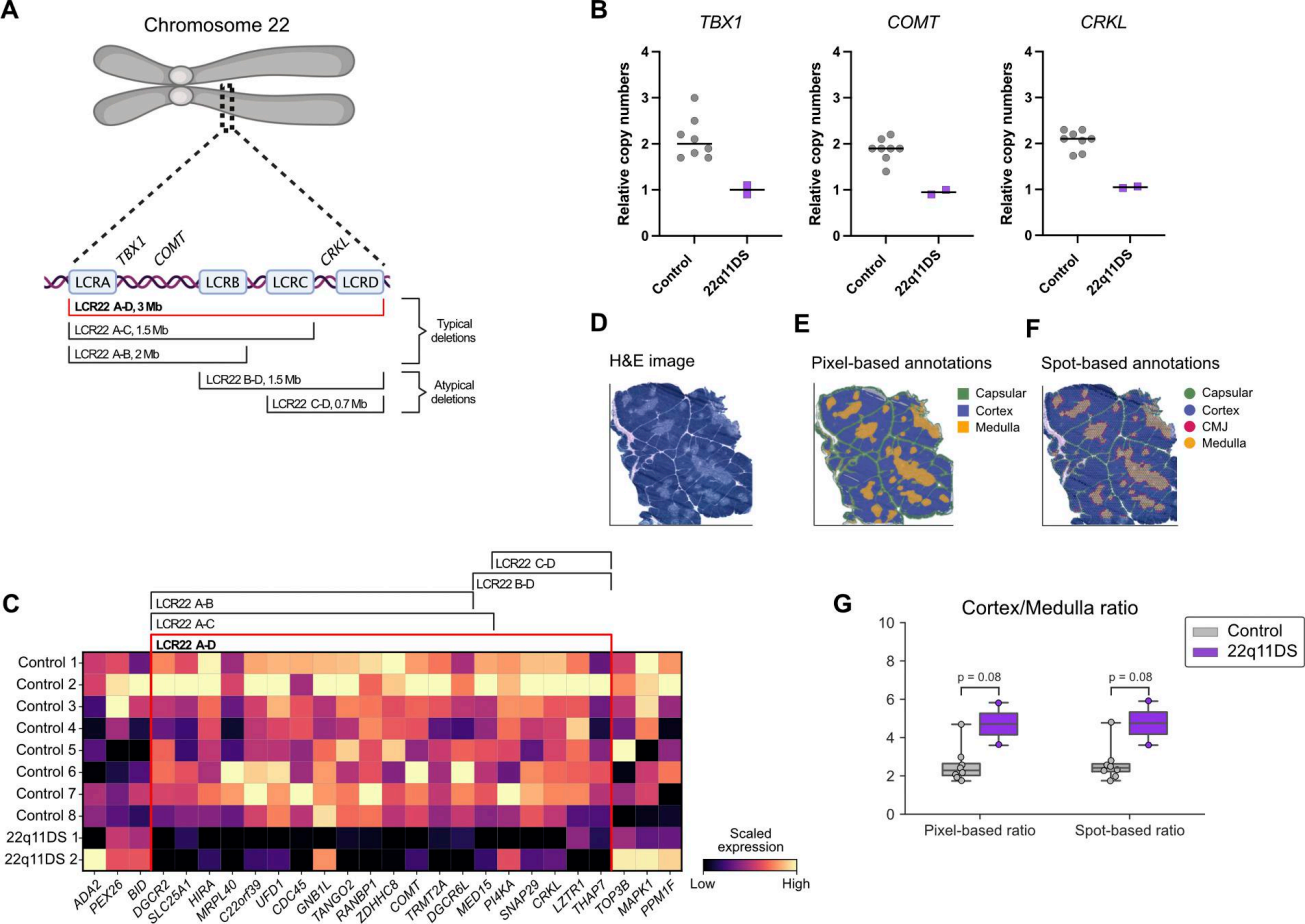
**Transcriptomic mapping confirms reduced gene expression in the LCR22A–D region and an increased corticomedullary ratio in 22q11DS thymus. (A)** Schematic overview of chromosome 22 with the most common deletions in the 22q11.2 region. Approximate locations of key genes within the 22q11.2 deletion region, *TBX1*, *COMT*, and *CRKL*, are marked. The LCR22A–D deletion present in the patient study cohort is highlighted in red. **(B)** Quantification of *TBX1*,* COMT*, and *CRKL* in thymic tissue using qPCR. Results are shown as relative gene copy numbers for each sample in controls (gray, *n* = 8) and 22q11DS patients (purple, *n* = 2). **(C)** Heatmap showing the Visium-derived expression of genes located within and flanking the 22q11.2 deletion region in controls (*n* = 8) and 22q11DS patients (*n* = 2). The expression of each gene is scaled using min-max scaling, where the purple color indicates low expression, and yellow indicates high expression. **(D)** H&E-stained section of a representative thymic tissue sample used for Visium spatial transcriptomics. **(E)** Pixel-based annotations in a representative thymic tissue sample. Tissue regions were annotated by combining gene expression–based information from Visium with manual curation of the H&E image to generate annotations: cortex (blue), medulla (orange), and capsule (green). **(F)** Annotation of Visium spots in a representative thymic tissue section. Visium spots were assigned to morphologically defined regions: capsular (green), cortex (blue), CMJ (pink), and medulla (orange). **(G)** Cortex-to-medulla ratios in controls and 22q11DS patients calculated using both pixel-based annotations and Visium spot-based annotations. Boxplots show cortex/medulla ratio distributions for controls (gray, *n* = 8) and 22q11DS patients (purple, *n* = 2). Differences between the groups were compared using two-sided Mann–Whitney U tests. H&E, hematoxylin and eosin.

The 22q11.2 deletion syndrome (22q11DS) is the most common microdeletion syndrome, with an estimated prevalence of 1 in 2,000–3,000 live births (1). It is caused by a hemizygous deletion in the 22q11.2 locus that can vary in size (0.7–3 Mb) and the number of included genes (∼10–55 genes) between individuals (Fig. 1 A) (2). Clinically, 22q11DS encompasses a broad spectrum of congenital anomalies, including cardiac outflow tract defects, palatal abnormalities, endocrine dysregulation, and thymic hypoplasia of variable severity (2). Expanded newborn screening programs have increased early identification of affected infants (3, 4). While a minority present with congenital athymia and a severe combined immune deficiency (SCID)–like phenotype requiring thymic transplantation, most exhibit T lymphopenia with persistent immunologic abnormalities and immune dysregulation (5, 6, 7, 8).

Both preclinical and human studies have identified multiple strategies to enhance thymopoiesis and thymic repair in settings of thymic insufficiency (9, 10, 11, 12, 13). However, translation to 22q11DS is constrained by limited characterization of which stromal, epithelial, and hematopoietic programs are dysregulated in the human 22q11DS thymus. Prior analyses of 22q11DS thymic tissue are scarce and largely based on histology and low-parameter imaging. They report architectural abnormalities including reduced medullary representation, specific changes in the medullary thymic epithelial cell (mTEC) compartment, and impaired early thymocyte development accompanied by low numbers of thymic and peripheral regulatory T cells (Tregs) (7, 14). Alterations in the B cell compartment have also been described, without clear association with thymic size or the degree of T cell lymphopenia (15, 16, 17).

The thymic hypoplasia in 22q11DS has classically been attributed to haploinsufficiency of the T-box transcription factor (*TBX1*), a gene located in the 22q11.2 locus (Fig. 1 A) (18), which regulates pharyngeal development and influences thymic epithelial differentiation via forkhead box N1 (FOXN1) and WNT-related pathways (19). Nonetheless, murine models support a broader stromal contribution. In *Tbx1* and *Crkl* haploinsufficient mice*,* impaired crosstalk between mesenchymal cells, epithelial cells, and thymocytes reshapes the thymic microenvironment (20). In *Tbx1*^neo/neo^ mice, increased deposition of collagen by mesenchymal cells restricts thymic growth and can be reversed by replacement with wild-type cells (21, 22).

Here, we have integrated spatial transcriptomics and spatial proteomics to map thymic architecture and cellular neighborhoods in 22q11DS compared with controls and identified dysregulated pathways in both lymphoid and stromal cell compartments in 22q11DS.

## Results

### Study participants

22q11DS patient 1 is a female who was born premature at 32 wk of gestational age, with a birth weight of 1,870 g. She had facial features typical for 22q11DS and presented with a congenital heart defect (truncus type 2, ventricular and atrial septal defects), hypocalcemia, and diaphragmatic hernia. Fluorescence in situ hybridization (FISH) testing at 2 wk of age established a diagnosis of 22q11DS. Surgical repair for the cardiac defect led to removal of a hypoplastic thymus, as part of a standard procedure to get access to the heart. At the time of diagnosis, her T cell count was slightly low for age (CD3 2.0 × 10^9^/L, CD4 1.6 × 10^9^/L, CD8 0.6 × 10^9^/L). Retrospective analysis of T cell receptor excision circles (TRECs) was within normal range (53 copies/μl). 22q11DS patient 2 is a male who was born full term at 40 wk of gestational age, with a birth weight of 3,445 g. Surgical repair of a tetralogy of Fallot at 1 mo of age led to removal of a grossly normal-appearing thymus. He later presented with undescended testis, renal agenesis, and velopharyngeal insufficiency leading to a diagnosis of 22q11DS by FISH testing at age 3 years. At the time of surgery, his T cell count was normal (CD3 2.8 × 10^9^/L, CD4 2.4 × 10^9^/L, CD8 0.6 × 10^9^/L). Retrospective analysis of TREC on the neonatal dried blood spot was within normal range (63 copies/μl). Control thymic samples were obtained from eight immunologically healthy children undergoing corrective heart surgery. The control cohort consisted of children aged 3–5 mo, with balanced representation of both sexes (*n* = 8, 50% females) (Table S1).

### A transcriptomic map confirms in situ downregulation of genes in the LCR22A–D region and reveals an increased corticomedullary ratio in the 22q11DS thymus

Deletion of the 22q11.2 region in the patients was confirmed using quantitative PCR (qPCR) on whole thymic tissue using TaqMan probes targeting genes *TBX1* and *COMT*, located in the proximal LCR22A–B region, and *CRKL*, located in the distal LCR22A–D region (Fig. 1 A). Both patients exhibited haploinsufficiency of all three genes (Fig. 1 B), consistent with the presence of the LCR22A–D deletion, which is observed in 85% of 22q11DS patients (2). While prior work suggests that spatial and transcriptomic regulation of thymic function in 22q11DS is impaired, the organizational and spatial changes in the 22q11DS thymus have not been thoroughly investigated. To address this, we generated a spatial transcriptomic atlas of the human thymus in 22q11DS using the Visium platform and integrated with CODEX (now Akoya PhenoCycler) analysis of tissue sections adjacent to the sections used for spatial transcriptomics as previously described (23, 24). To assess the usability of this approach in 22q11DS, we assessed gene expression in the deleted locus, confirming in situ downregulation of genes located in the LCR22A-D region also on the RNA level (Fig. 1 C).

**Figure S1.**
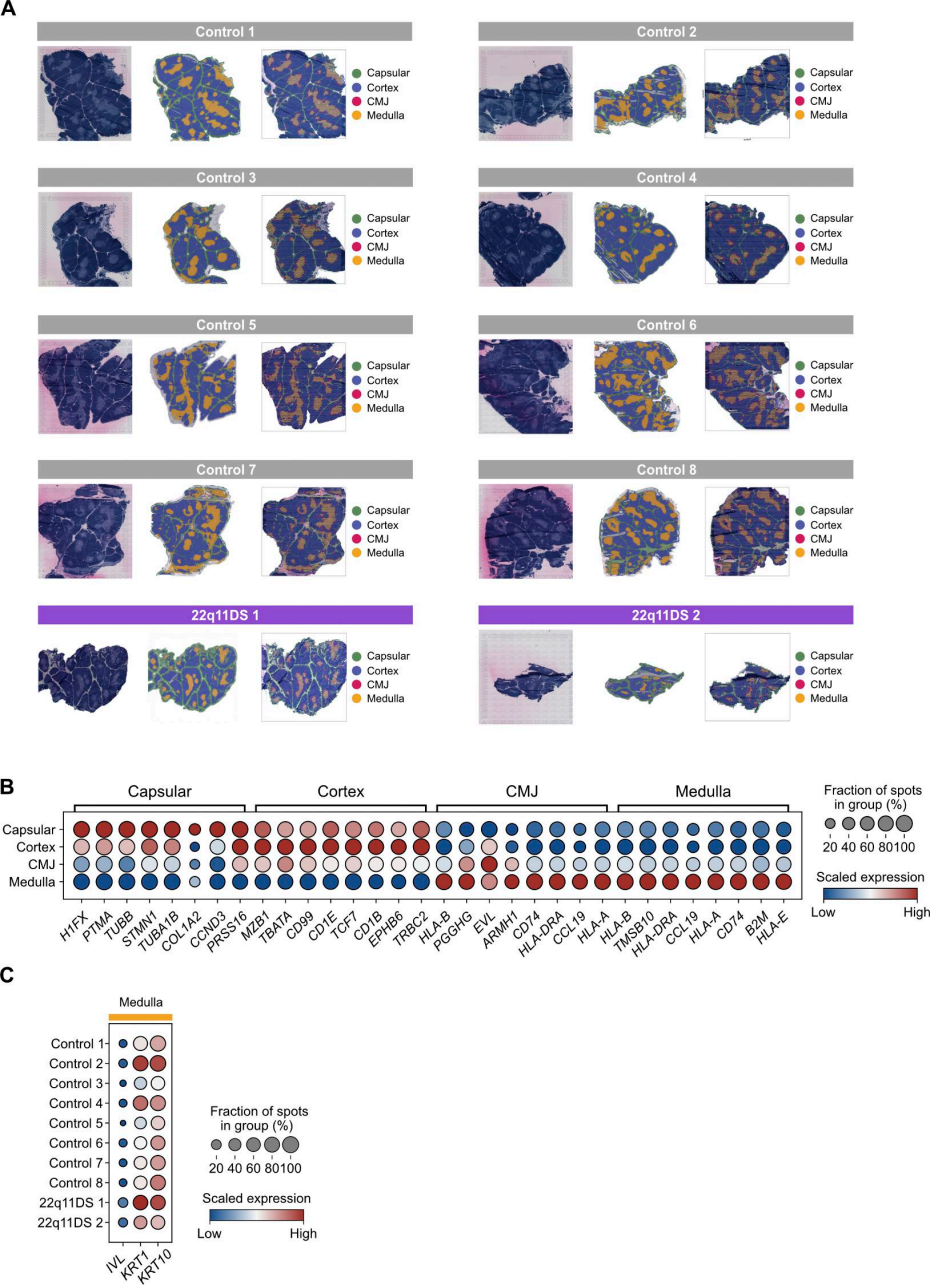
**Annotation of thymic tissue regions and validation by marker gene expression. (A)** H&E stainings, pixel-based annotations, and spot-based annotations of all thymic samples used in this study. Tissue regions were annotated by combining gene expression–based information from Visium with manual annotation of the H&E image. Visium spots were assigned to anatomically defined regions: capsular, cortex, CMJ, and medulla. The color of spots indicates region identity. **(B)** Dotplot showing the expression of marker genes in annotated regions. The expression of each gene is scaled using min-max scaling, where the blue color indicates low relative expression, and the red color indicates high relative expression. **(C)** Dotplot showing the expression of Hassall’s corpuscle marker genes *IVL*, *KRT1*, and *KRT10* in medullary spots. Scaled gene expression is shown, with blue indicating low relative expression and red indicating high relative expression. H&E, hematoxylin and eosin.

Following data integration, quality control, and filtering of spatial transcriptomics data, our first approach was to annotate the thymic tissue architecture. Anatomical regions—capsular, cortex, corticomedullary junction (CMJ), and medulla—were defined using TissueTag (25), and assigned to each Visium spot (Fig. 1, D–F and Fig. S1 A). The annotations were further validated by examination of differentially expressed marker genes (Fig. S1 B). This analysis suggested an increased corticomedullary ratio in the 22q11DS thymus (Fig. 1 G), when assessing both pixel-based and spot-based annotations (P = 0.08) consistent with previous reports (14). Identification of Hassall’s corpuscles by transcriptomic analysis is inherently challenging, due to their very low overall transcriptional activity. However, no difference was seen between 22q11DS and controls regarding the frequency of Hassall’s corpuscles, as defined by the expression of *IVL*, *KRT1*, and *KRT10* (Fig. S1 C).

### Altered frequencies of fibroblasts, B cells, Tregs, and mTEC subsets in the 22q11DS thymus

To identify cell types that were underlying the morphological changes, we performed deconvolution using publicly available single-cell RNA sequencing (scRNA-seq) data (26) using the Cell2location package for Python (27) (Table S2) enabling us to predict the contribution of 35 different cell types in every Visium spot and their relationship to colocalization patterns and cell-specific gene expression.

**Figure 2.**
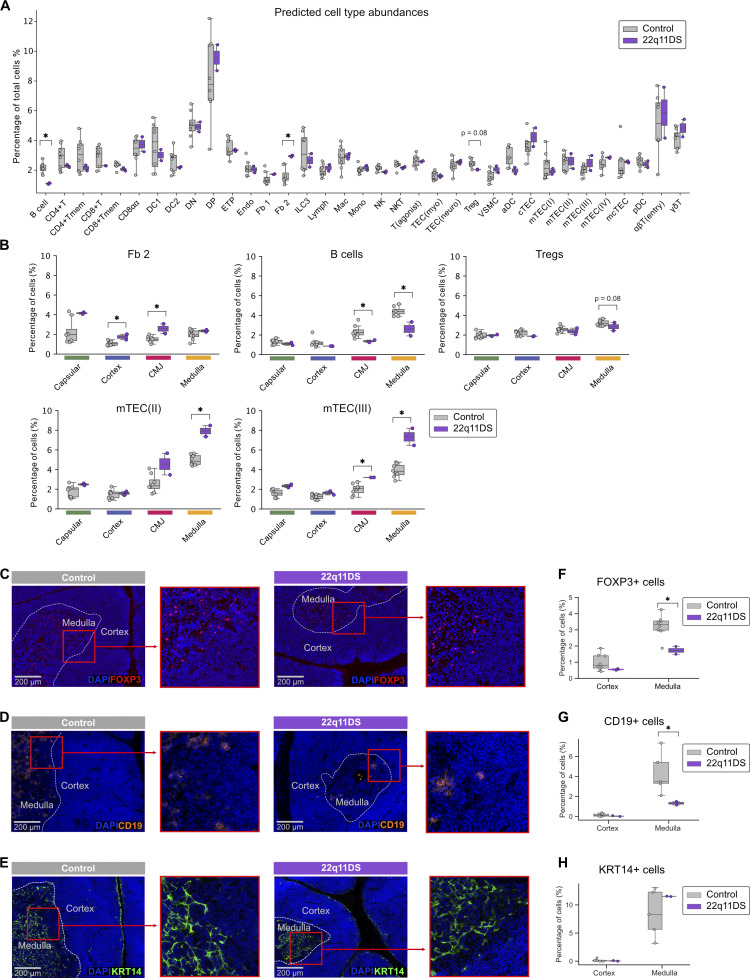
**Altered cellular composition of the 22q11DS thymus. (A)** Barplots showing predicted cell-type abundance. Cell-type abundance is shown as mean percentages of total thymic cells. Controls (*n* = 8) are shown in gray, and 22q11DS patients (*n* = 2) are shown in purple. Differences in cell-type abundance were assessed using two-sided Mann–Whitney U tests. **(B)** Predicted abundance of Fibroblast type 2 (Fb 2), B cells, regulatory T cells (Tregs), medullary epithelial cells, subtype II (mTEC(II)), and medullary epithelial cells, subtype III (IImTEC(III)) in 22q11DS patients (purple, *n* = 2) and controls (gray, *n* = 8) shown as mean percentages of total cells in each annotated anatomical region (* indicates P <0.05). **(C–E)** CODEX immunofluorescence staining of Tregs (C), B cells (D), and Keratin 14 positive medullary epithelial cells (KRT14^+^ mTECs) (E) in representative thymic tissue samples from 22q11DS patients and controls. Cell nuclei are stained using DAPI (blue). Tregs are stained using anti-FOXP3 antibody (red), B cells are stained using anti-CD19 antibody (orange), and KRT14^+^ mTECs are stained using anti-KRT14 (green). **(F–H)** Boxplot showing mean percentages of Tregs (F), B cells (G), and KRT14^+^ mTECs (H) in cortex and medulla in the CODEX data. Image processing and segmentation were performed in QuPath, followed by Gaussian mixture rescaling and cell phenotyping in Python using Scimap’s phenotype_cells function. Differences in cell abundance between 22q11DS patients (purple, *n* = 2) and controls (gray, *n* = 8 for Tregs, *n* = 5 for B cells, and *n* = 5 for KRT14^+^ mTECs) were assessed using two-sided Mann–Whitney U tests.

**Figure S2.**
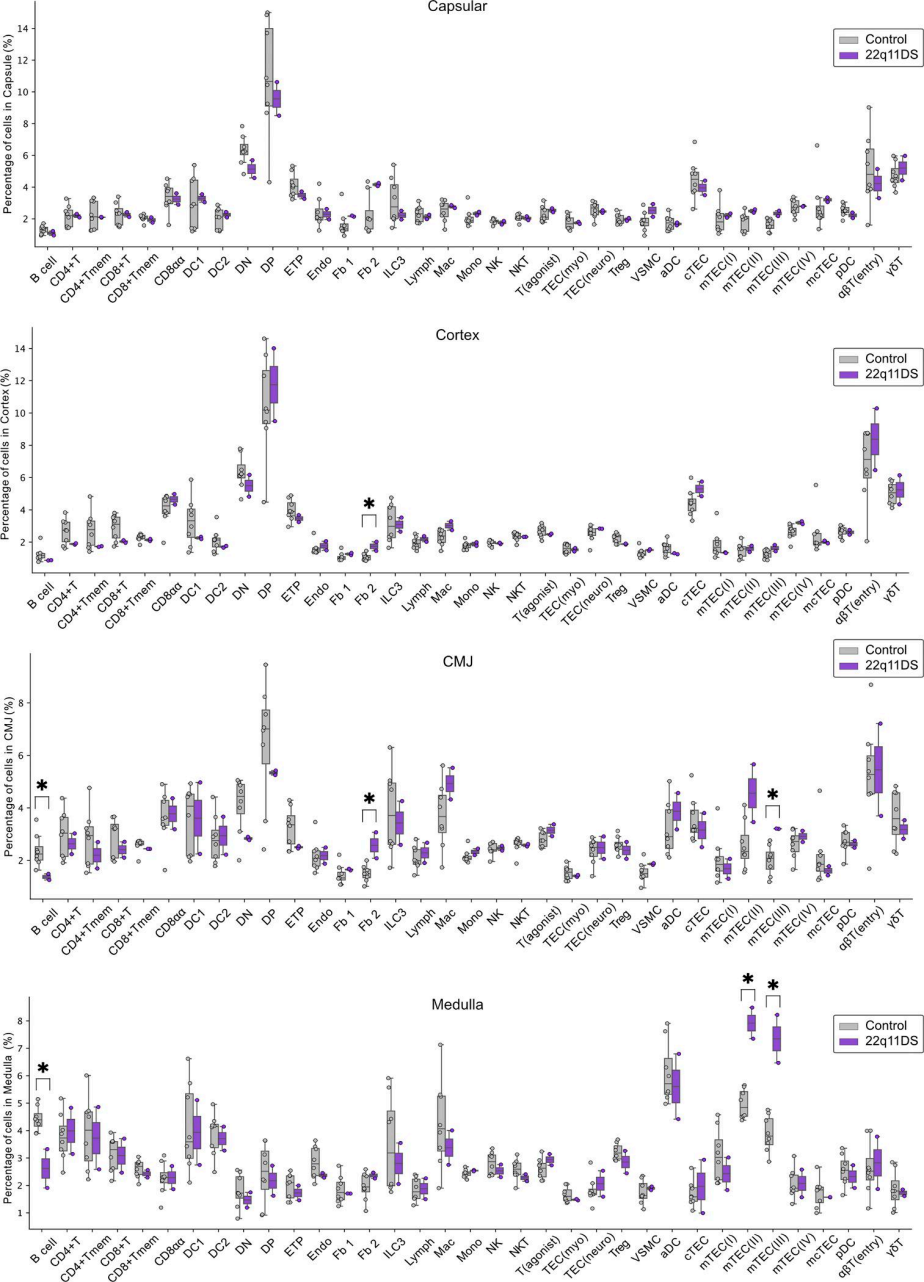
**Barplots showing predicted cell-type abundance in the capsule, cortex, CMJ, and medulla.** Cell abundance is shown as mean percentages of total cells in each region. Controls (*n* = 8) are shown in gray, and 22q11DS patients (*n* = 2) are shown in purple. Differences in cell-type abundance were assessed using two-sided Mann–Whitney U tests (* indicates P <0.05). Abbreviations for cell-type annotations are provided in Table S2.

When comparing overall cell-type abundance between 22q11DS patients and controls, we saw that the patients showed an increased frequency of fibroblast type 2 (Fb 2) and decreased frequency of thymic B cells (Fig. 2 A). In addition, a trend toward a decreased frequency of Tregs in 22q11DS was noted (P = 0.088) (Fig. 2 A). Further analyses of cell abundance, separately in the different morphological compartments, were performed (Fig. S2). This showed that differences in Fb 2 abundance were restricted to the CMJ and cortex, and as expected that the greatest differences in B cell and Treg abundance were seen in the CMJ and medulla (Fig. 2 B). Interestingly, the separate analysis of the different compartments also identified markedly increased frequencies of mTEC(II) and mTEC(III) in 22q11DS in the medulla and CMJ (Fig. 2 B).

Decreased numbers of thymic B cells and Tregs, and a trend toward an increased frequency of KRT14^+^ mTECs, in the medulla of 22q11DS patients were confirmed on the protein level by mapping of CD19^+^, FOXP3^+^, and KRT14^+^ cells in adjacent tissue with CODEX (Fig. 2, C–H).

### The 22q11DS thymus shows alterations in extracellular matrix–related gene expression and decreased inflammatory signaling

**Figure 3.**
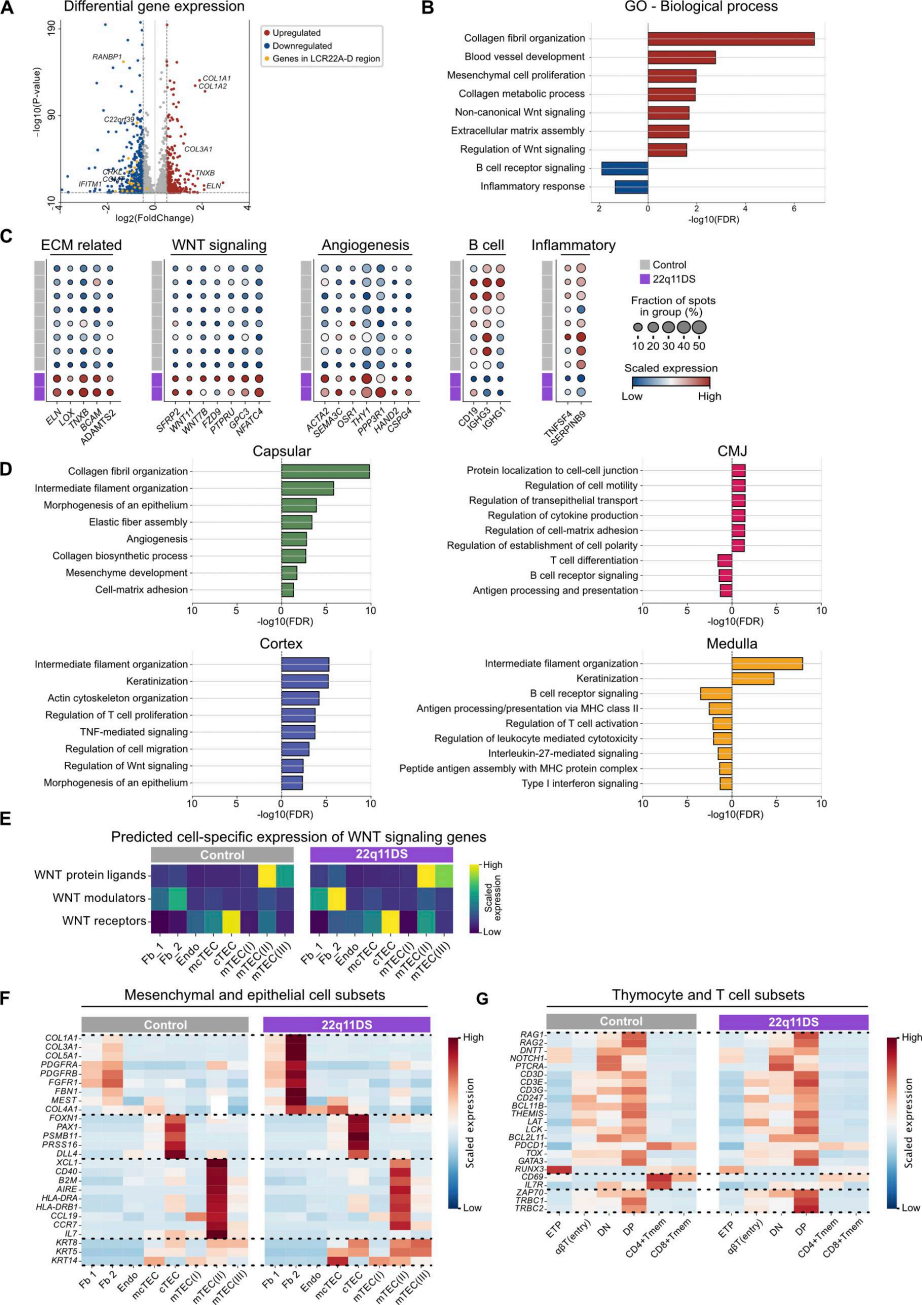
**Differently expressed genes highlights altered extracellular matrix pathways and reduced inflammatory signaling in the 22q11DS thymus. (A)** Volcano plot of DEGs in 22q11DS patients (*n* = 2) compared with controls (*n* = 8). Comparison between the groups was performed using the two-sided Wilcoxon rank-sum test with the Benjamini–Hochberg correction for multiple testing. Significant DEGs (adjusted P <0.05) are shown in red (upregulated) and blue (downregulated). Genes located in the 22q11.2 deletion region (LCR22A–D) are marked in yellow. **(B)** Histogram showing up- and downregulated GO terms in the 22q11DS thymus. GO enrichment analyses were performed on significant DEGs in Fig. 3 A, using the PANTHER classification system. Significantly upregulated GO terms (adjusted P value, FDR <0.05) point to the right in red, and significantly downregulated GO terms point to the left in blue. **(C**) Dotplots showing the expression in the whole thymus tissue of selected genes associated with significantly enriched GO terms in Fig. 3 B. The expression of each gene is scaled using min-max scaling, where the blue color indicates low expression, and the red color indicates high expression. The dot size represents the proportion of total Visium spots in which the gene is expressed. **(D)** Histograms showing significantly up- and downregulated GO terms per annotated morphological region in 22q11DS patients compared with controls. GO enrichment analyses were performed on the significant DEGs in each region: capsule, cortex, CMJ, and medulla. Significantly upregulated GO terms (adjusted P value, FDR <0.05) point to the right, and significantly downregulated GO terms point to the left. The colors of the bars indicate region identity. **(E)** Heatmap showing the predicted cell type–specific expression of WNT signaling components in 22q11DS patients and controls. Gene expression is presented as scaled module scores where WNT protein ligands are represented by genes *WNT5* and *WNT4*, WNT modulators by *SFRP2*,* SFRP4*,* SFRP1*,* DKK1*, and *GPC3*, and WNT receptors by *FZD9*, *FZD4*,* FZD5*,* FZD8*,* ROR2*, and *RYK.* The predicted cell type–specific expression of each gene is shown in Fig. S2 A. **(F)** Heatmap showing the expression of selected genes in mesenchymal and epithelial cell subsets in 22q11DS patients (*n* = 2) and controls (*n* = 8). For visualization, each gene was scaled using the Z-score. The blue color indicates low relative expression, and the red color indicates high relative expression. Dotted lines separate gene sets: mesenchymal transcripts, cortical thymic epithelial cells (cTEC)-associated genes, genes associated with selection processes, and genes encoding cytokeratins. **(G)** Heatmap showing the expression of selected genes in thymocyte and T cell subsets in 22q11DS patients (*n* = 2) and controls (*n* = 8). For visualization, each gene was scaled using the Z-score. The blue color indicates low relative expression, and the red color indicates high relative expression. Dotted lines separate gene sets involving early T cell activation and T cell receptor signaling.

When comparing the overall transcriptome in the 22q11DS thymus to controls using differently expressed genes (DEG) analysis, we identified several significantly enriched genes outside the 22q11.2 region, indicating broader downstream alterations caused by the haploinsufficiency (Fig. 3 A). To connect the observed gene expression changes to biological pathways, we performed gene ontology (GO) enrichment analysis using the identified DEGs. The result revealed an overall upregulation of genes involved in collagen formation, extracellular matrix (ECM) assembly, endothelial cell proliferation, and WNT signaling (Fig. 3 B). Most strikingly, genes that contribute to the organization, elasticity, and cross-linking of the ECM, such as lysyl oxidase (*LOX*), tenascin-X (*TNXB*), and elastin (*ELN*), were highly upregulated in the 22q11DS thymus (Fig. 3 C). Furthermore, 22q11DS thymus showed increased expression of genes involved in vascular development and mesenchymal–endothelial crosstalk such as actin α 2 (*ACTA2*) and semaphorin-3C (*SEMA3C*) together with WNT signaling–related genes such as *SFRP2*,* WNT11*, and *NFATC4*. We also noted downregulation of B cell–related genes such as immunoglobulin genes together with genes involved in immune activation and inflammatory signaling such as *TNFSF4* and *SERPINB9* (Fig. 3 C).

To connect these changes to compartmental signaling, we performed DEG and GO-term analyses per compartment, revealing that the upregulation of collagen, ECM, and mesenchymal/endothelial cell signaling was predominantly driven by the capsular regions (Fig. 3 D). In the 22q11DS thymus, increased WNT signaling in the cortical area, and increased keratinization both in the cortical and in the medullary compartments, was noted. As expected, the downregulation of B cell receptor signaling pathway was primarily found in the CMJ and medullary regions (Fig. 3 D).

**Figure S3.**
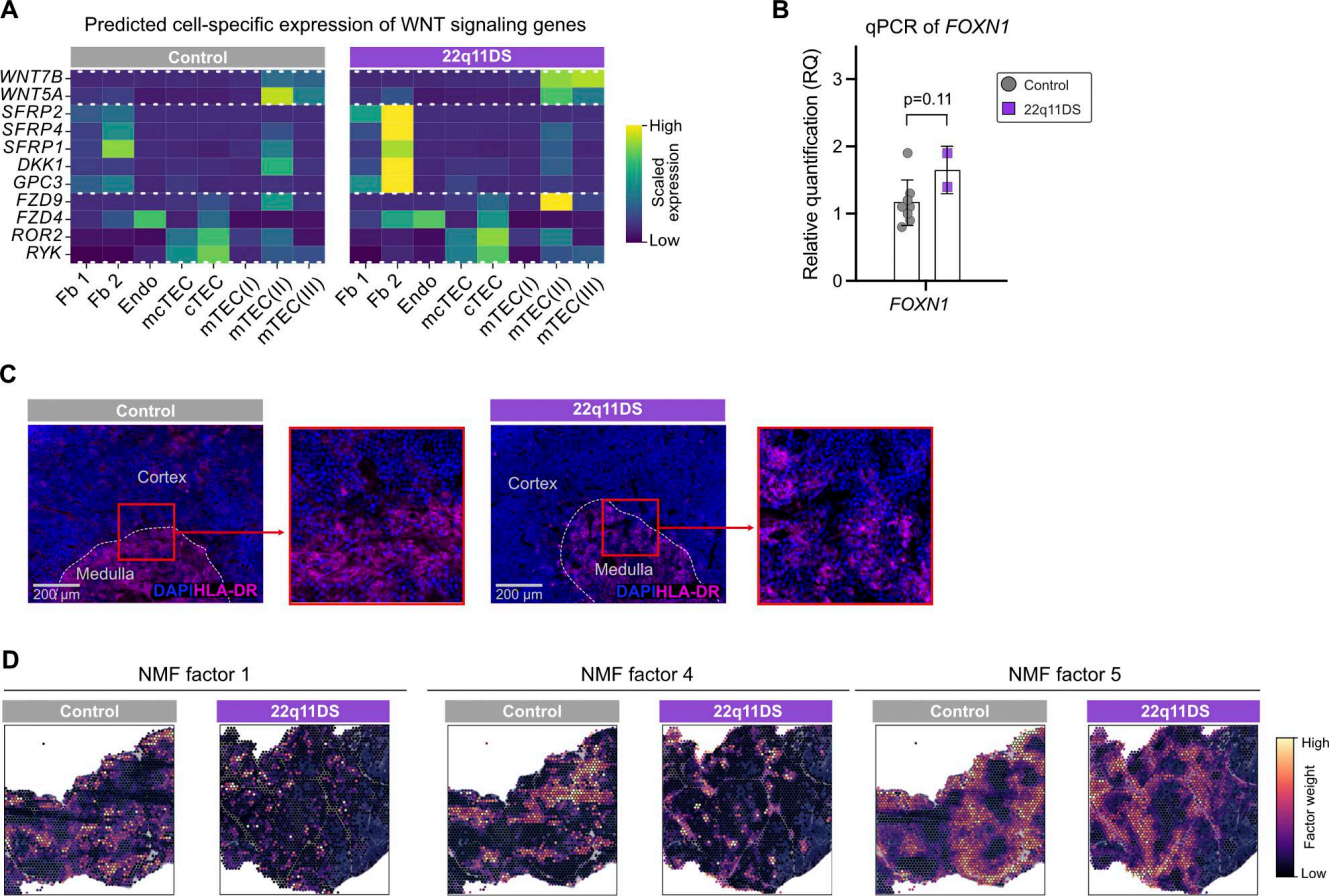
**Alterations in WNT signaling, FOXN1 expression, and spatial niche organization in the 22q11DS thymus. (A)** Heatmap showing the predicted cell type–specific expression of genes involved in WNT signaling in 22q11DS patients and controls. For visualization, each gene was scaled using Z-score. The blue color indicates low relative expression, and the yellow color indicates high relative expression. Dotted lines separate WNT ligands, WNT modulators, and WNT receptors. **(B)** Barplot showing results from qPCR-based quantification of *FOXN1* transcripts in 22q11DS patients and controls. qPCR analysis was performed on fresh-frozen whole thymic tissue from controls (gray, *n* = 8) and 22q11DS patients (purple, *n* = 2) with TaqMan probes targeting *FOXN1*. Results are presented as relative quantification (RQ: 2^(−ΔΔCt)). **(C)** CODEX immunofluorescence staining of anti-HLA-DR (pink) and DAPI (blue) in representative thymic tissue samples from 22q11DS patients and controls. **(D)** NMF factors 1, 4, and 5 are plotted spatially in representative tissue samples from 22q11DS patients and controls. The color of each spot indicates factor weight, with purple representing low relative weight and yellow representing high relative weight.

WNT signaling is known to be important for thymic organogenesis and function by guiding thymic epithelial cell (TEC) differentiation and regulating cell migration, tissue organization, and compartmentalization (28). When analyzing predicted cell-specific expression, we observed that WNT modulators were mainly expressed by fibroblasts, whereas receptors and ligands were highly expressed in TECs (Fig. 3 E and Fig. S3 A). This suggests a disturbed interplay in WNT signaling between mesenchymal cell types and the TECs in the 22q11DS thymus. A key regulator of cortical epithelial differentiation is the transcription factor *FOXN1*, which showed increased expression in 22q11DS cTECs compared with controls (Fig. 3 F), supported by results from qPCR analysis (Fig. S3 B). Together with increased levels of *FOXN1*, the cTEC compartment also showed upregulation of genes such *PSMB11* and *PRSS16* coinciding with a decreased expression of *IL7*, which is essential for early thymocyte survival and proliferation (Fig 3 F). In the mTEC compartment, the increased frequencies of mTEC(II) and mTEC(III) in 22q11DS are accompanied by an overall downregulation of genes involved in antigen presentation and negative selection, for example, *XCL1*,* AIRE*, and HLA-DR molecules (Fig. 3 F and Fig. S3 C). Both cTECs and mTECs show an upregulation of signature cytokeratin genes *KRT5*,* KRT8*, and *KRT14*.

In-depth analysis of the stromal, endothelial, and epithelial predicted gene expression demonstrated that the largest differences between 22q11DS patients and controls were observed in the mesenchymal subsets and predominantly in fibroblasts (Fig. 3 F). However, predicted cell type–specific gene expression in thymocytes revealed no substantial differences between patients and controls when looking at recombination and selection markers such as *RAG1*,* RAG2*,* THEMIS*,* CD99*, with only changes in expression of *CD69*, and *IL7R* in the 22q11DS CD8^−^ and CD4-positive cells (Fig. 3 G). Altogether, this indicates that the 22q11DS thymus is characterized by moderate changes in epithelial cells and maintained thymocyte development, coinciding with broad changes in mesenchymal cells and ECM expression.

### Fibroblast-derived collagens are upregulated in the 22q11DS thymus

**Figure 4.**
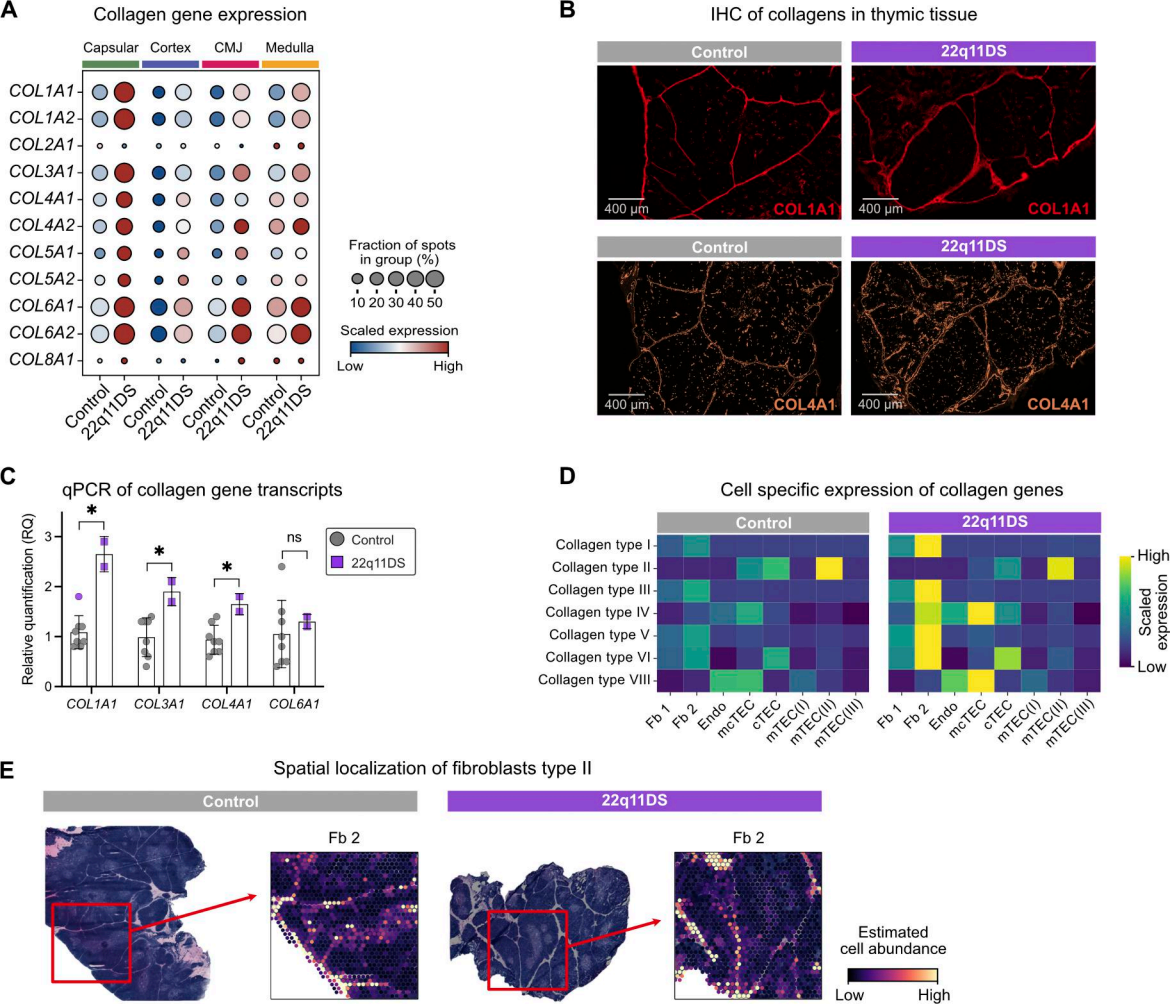
**Increased expression of fibroblast-derived collagens in the 22q11DS thymus. (A)** Dotplot of collagen gene expression in each region in 22q11DS patients and controls. Average gene expression values were plotted using log-normalized raw counts. For visualization, the expression of each gene was scaled using min-max scaling. The blue color indicates low relative expression, and the red color indicates high relative expression. The dot size represents the proportion of total Visium spots in which the gene is expressed. **(B)** Representative immunofluorescence stainings of collagen type I and IV in control and 22q11DS thymic sections. **(C)** Barplots showing results from qPCR-based quantification of collagen gene transcripts in 22q11DS patients (purple, *n* = 2) and controls (gray, *n* = 8). qPCR analyses were performed on the fresh-frozen whole thymic tissue. Results are presented as relative quantification (RQ: 2^^(−ΔΔCt)^). **(D)** Heatmap of predicted cell type–specific expression of collagens. Gene expression is presented as scaled module scores, where collagen type I is represented by genes *COL1A1* and *COL1A2*, collagen type II by *COL2A1*, collagen type III by *COL3A1*, collagen type IV by *COL4A1* and *COL4A2*, collagen type VI by *COL6A1* and *COL6A2*, and collagen type VIII by *COL8A1.***(E)** Visualization of estimated Fb 2 abundance in spatial coordinates in representative tissue samples from 22q11DS patients and controls. Dark purple indicates low abundance, and yellow indicates high abundance *P value < 0.05; ns, not significant.

When looking more closely into the expression of collagens, we noted that several types of collagens were upregulated in the 22q11DS thymus (Fig. 4 A). While ECM proteins are known to be important in multiple lymphoid organs, their role in the human thymus and how they are modulated in disease settings remain poorly understood. Collagens are major structural components of the thymic ECM. Fibrillar collagens (types I, II, and III) form connective scaffolds within the capsule and interlobular septae, providing mechanical stability defining thymic lobular architecture (29). Network-forming collagens include type IV, the principal structural component of basement membranes that delineate epithelial and vascular interfaces, and type VIII, a short-chain network-forming collagen associated with vascular endothelium and ECM remodeling (20). Collagen type VI is a fibroblast-associated ECM component expressed by capsular and medullary thymic fibroblast subsets and contributes to stromal integrity and compartmentalization. Type I, III, IV, V, VI, and VIII collagens, such as *COL1A1*,* COL3A1*,* COL4A1*,* COL5A1*,* COL6A1*, and *COL8A1*, were strongly upregulated in the 22q11DS thymic capsule, although increased expression was also seen in other thymic compartments (Fig. 4 A). The spatial locations of collagens type I and IV were confirmed using immunofluorescence staining of thymic tissue from controls and 22q11DS using anti-COL1A1 and anti-COL4A1 antibodies (Fig. 4 B). To validate these transcriptomic differences in collagen expression, we performed qPCR on whole thymus tissue, which confirmed an upregulation of *COL1A1*,* COL3A1*, and *COL4A1* expression in the 22q11DS thymus (Fig. 4 C). When plotting the estimated expression of collagen genes per cell type, we confirmed that fibroblast subsets were the main producers of collagen (Fig. 4 D). We could identify both perilobular fibroblasts (Fb 1), characterized by the expression of *COLEC11*,* C7*, and *GDF10*, and capsular/interlobular fibroblasts (Fb 2) characterized by the expression of *PI16*, *FN1*, and *FBN1* (26). Collagens type I, III, and V were predicted to be highly expressed in Fb 2, whereas collagens type II, IV, VI, and VIII showed a broader expression pattern and predicted expressions were also seen in endothelial and epithelial cell subsets (Fig. 4 D). Next, to assess the spatial localization of the collagen-producing fibroblasts, we mapped Fb 2 by spatial projection of the cell abundance matrix revealing that Fb 2 is primarily found in the capsular region of the thymus (Fig. 4 E). Taken together, this indicates an increased expression of collagens by fibroblasts with retained spatial localization in 22q11DS.

### Colocalization analysis reveals alterations in spatial niches in the 22q11DS thymus

**Figure 5.**
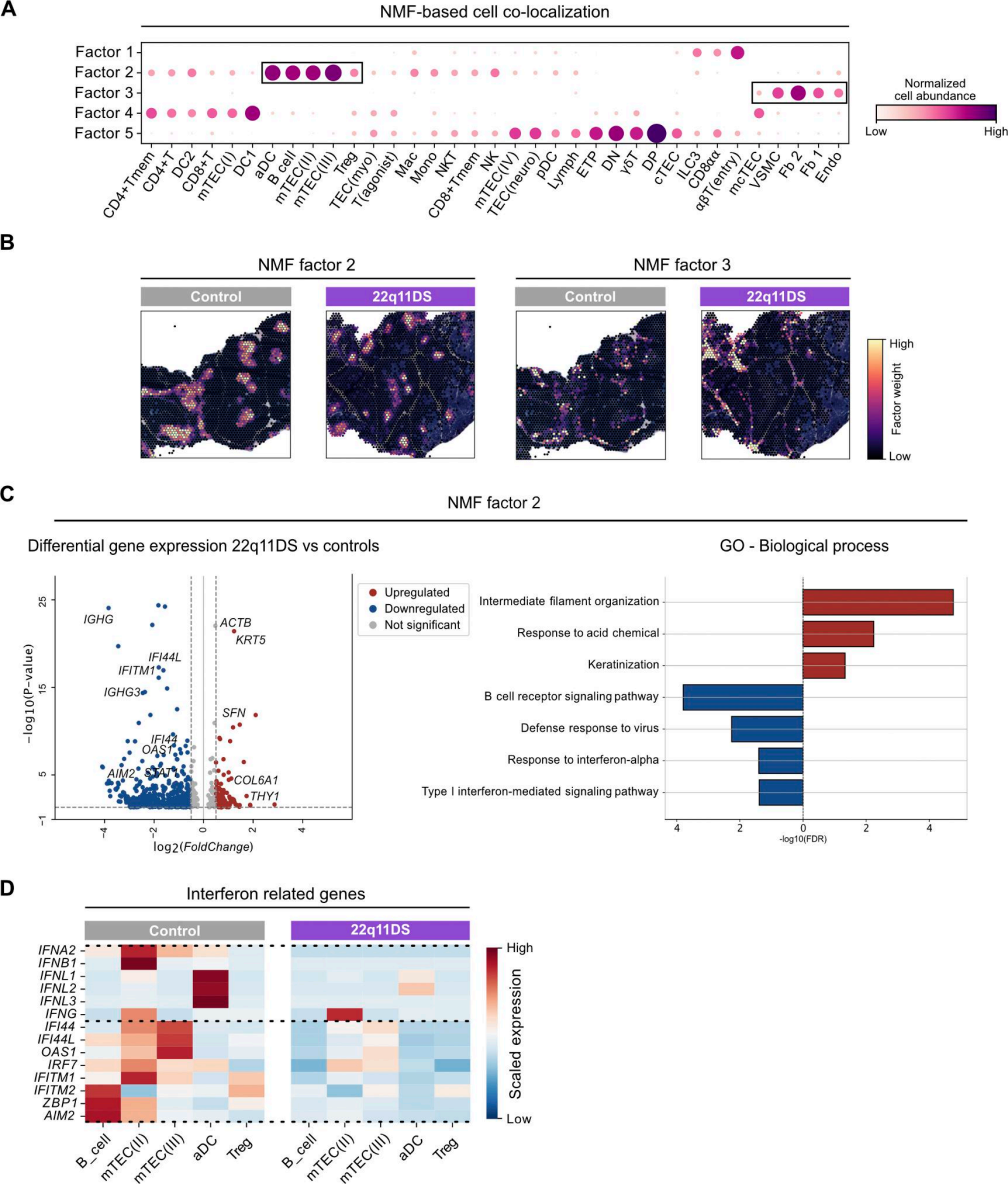
**Alterations in spatial niches in the 22q11DS thymus. (A)** Identification of spatially colocalized cellular compartments using NMF. The results are shown as a dotplot with cell-type abundance (columns) across NMF factors (rows). Each factor represents a spatially co-occurring group of cell types. The dot size and dot color correspond to NMF weights and normalized cell-type abundance. Boxes highlight colocalized groups of cells in factors 2 and 3. **(B**) NMF factor 2 and 3 plotted spatially in representative tissue samples from 22q11DS patients and controls. The color of each spot indicates factor weight, with purple representing low relative weight and yellow representing high relative weight. Spatial locations of NMF factors 1, 4, and 5 are shown in Fig. 4 A. **(C)** Volcano plot of DEGs in NMF factor 2 in 22q11DS patients (*n* = 2) compared with controls (*n* = 8). Comparison between the groups was performed using the two-sided Wilcoxon rank-sum test with Benjamini–Hochberg correction for multiple testing. Significant DEGs (adjusted P <0.05) are shown in red (upregulated) and blue (downregulated). Histogram showing up- and downregulated GO terms in 22q11DS NMF factor 2. GO enrichment analyses were performed on the significant DEGs in Fig. S3 A using the PANTHER classification system. Significantly upregulated GO terms (adjusted P value, FDR <0.05) point to the right in red, and significantly downregulated GO terms point to the left in blue. **(D)** Heatmap showing the expression of interferon-related genes in selected cell-type subsets in 22q11DS patients (*n* = 2) and controls (*n* = 8). Each gene was scaled using the Z-score. The blue color indicates low expression, and the red color indicates high expression. The dotted line separates gene sets including interferons and interferon-stimulated genes. Abbreviations for cell-type annotations are provided in Table S2.

To further define colocalization and predict potential cellular interactions, we applied nonnegative matrix factorization (NMF) to our estimated spatial cell abundance matrix. NMF is a dimensionality reduction technique that decomposes data into latent factors, where each factor captures a spatially co-occurring group of cell types. Five distinct factors were identified, with factor 3 showing colocalization of Fb 1 and Fb 2 with endothelial cells, vascular smooth muscle cells, and bipotent TEC progenitor cells (mcTECs), indicative of localized mesenchymal–endothelial cell interactions (Fig. 5 A). Spatial proximity could also be seen between B cells, Tregs, mTEC(II), mTEC(III), and activated dendritic cells (aDCs), which were all represented in factor 2 (Fig. 5 A), indicating a potential spatial cluster close to Hassall’s corpuscles in the deep medulla (Fig. 5 B) (25). Differently expressed genes analysis of NMF factor 2 comparing 22q11DS and controls revealed reduced expression of genes associated with interferon signaling pathways (Fig. 5 C). When assessing the cell-specific gene expression of interferon-related genes, we discovered that 22q11DS mTEC(II) expressed lower levels of type I interferons and aDCs expressed lower levels of type III interferons (30, 31, 32). Interferon-stimulated genes, such as *IFI44*,* IFITM1*,* IFITTM2*, and *OAS1*, were lower in mTEC(II), mTEC(III), B cells, and Tregs in the 22q11DS compared with controls (Fig. 5 D). Together, this suggests that structural alterations in stromal cells coincide with alterations in mature cell types and changes in inflammation-associated markers in the thymus in 22q11DS patients.

## Discussion

In this study, we reveal pronounced alterations in the architecture and transcriptional composition of the 22q11DS thymus using spatial transcriptomics with paired high-resolution imaging.

Our current work on the human 22q11DS thymus confirms previous findings from mouse models (21, 22) and provides additional novel insights into the alterations in the mesenchymal cell compartment, with increased deposition of collagens and ECM proteins, together with aberrant WNT signaling. Supported by these previous studies, we speculate that increased collagen production by mesenchymal cells may cause growth restriction in thymic lobes, potentially by thickening the outer fibrous layer of the thymus and decreasing capsule elasticity. Aberrant WNT activity in the mesenchymal compartment could lead to disrupted cell–cell interactions and impaired endothelial and epithelial developmental processes.

DEG analysis identified enrichment of genes associated with multiple pathways critical for thymic development and function, e.g., angiogenesis. Upregulation of genes involved in blood vessel development, such as *ACTA2*,* THY1*, *SEMA3C*, and* SPG4*, was seen in the 22q11DS thymus. We also saw increased expression in fibroblasts of molecules important for the support and differentiation of vascular endothelial cells such as *COL4A1*,* FBN1*, and *FGFR1*, along with dysregulated WNT signaling activity. The strong spatial correlation of mesenchymal and endothelial cell subsets further suggests that the altered expression of these molecules in fibroblasts also affects the vasculature in the 22q11DS thymus. Prior work has highlighted the critical role of mesenchymal–endothelial crosstalk in vascular developmental processes. Studies of blood vessel organoids using induced pluripotent cells from 22q11DS patients showed that 22q11DS organoids grew smaller than controls, had increased levels of both collagens and ECM proteins, and presented with diminished stability of the vascular network (33, *Preprint*). While our findings provide additional support for these observations, a detailed investigation of this is beyond the scope of the present study.

*TBX1*, one of the most thoroughly studied genes associated with 22q11DS, is known to play a role in both formation and positioning of the developing thymus, as well as the differentiation of the thymic stroma by regulating key transcription factors such as *FOXN1*. Retraction of *TBX1* is needed for the expression of *FOXN1*, which in turn is required for the differentiation of TECs (19). In line with this, the cTEC compartment in the 22q11DS thymus showed upregulated expression of *FOXN1*, as well as *FOXN1* target genes *PSMB11* and *PRSS16*. The timing and dosage of *FOXN1* expression have been shown to impact both stromal cell maturation and cortex–medulla compartmentalization (34, 35), which suggests that the increased *FOXN1* expression in the cTEC compartment may contribute to the increased cortex/medulla ratio in the 22q11DS thymus.

*TBX1* haploinsufficiency has also been linked to dysregulation of the WNT signaling pathway. Mouse studies have established that Tbx1 is important for both canonical and noncanonical Wnt signaling, which in turn is important for the establishment and positioning of organs, such as the thymus and the heart, as well as the activity and function of thymic mesenchymal cells (36, 37), emphasizing the importance of the sophisticated interplay between *TBX1*,* FOXN1*, and WNT signaling in the developing thymus.

Within the mTEC compartment, we observed an altered cortex/medulla ratio in 22q11DS compared with controls, with a lower representation of the medullary compartment. This is consistent with previous reports and has been suggested to reflect a delayed thymic maturation in 22q11DS (14). Interestingly, the reduced representation of the medullary compartment was accompanied by increased frequencies of mTEC(II) and mTEC(III) subsets within the medulla. We also noted lower expression levels of MHC class II–related genes and genes involved in negative selection processes, while the expression of cytokeratin genes was increased in 22q11DS. A speculation is that the morphological and functional alterations in the medullary epithelial cell compartment of the 22q11DS thymus impair central tolerance induction and contribute to the increased risk of autoimmune manifestations in 22q11DS patients (38, 39), but that remains to be elucidated.

We replicated, both on the transcriptome and on the protein level, the earlier observation of reduced numbers of thymic Tregs in 22q11DS (14). This may be a consequence of a perturbed medullary epithelial compartment, with alterations that include reduced expression of *IL7*, which is important for the viability of thymocytes and generation of Tregs. Interestingly, based on both Visium and CODEX data, we also observed markedly reduced numbers of medullary B cells in 22q11DS. Spatial clustering analysis by NMF showed that B cells and Tregs were colocalized, together with mTEC(II), mTEC(III), and aDCs, in a spatial niche, deep in the medulla, which has been identified earlier (25).

Various functions have been attributed to thymic B cells, especially related to self-antigen presentation and negative selection (40). In mouse models, interferon-stimulated B cells have also been shown to be of importance for thymic Treg generation (41), which led us to analyzing interferon-related gene signatures in the colocalized B cells, Treg, mTEC(II), mTEC(III), and aDCs. This revealed alterations in the 22q11DS thymus with reduced expression of interferon type I by mTEC(II) and interferon type III by aDCs, accompanied by the lower expression of interferon-stimulated genes in both B cells, mTEC(II), and mTEC(III). Interestingly, our data suggest that in human thymus, aDCs seem to be the main producers of type III interferons in the thymic medulla as compared to mouse where mTECs have been identified as the prime source of interferon type III (41). The results show that the inflammatory milieu in this spatial and functional niche, which is of importance for Treg formation, is impaired in 22q11DS. The low numbers of interferon-primed inflammatory B cells observed in 22q11DS may negatively influence Treg formation and contribute to low Treg numbers both in the thymus and in the periphery.

Spatial analyses of tissue sections, as compared to analyses of solubilized cells, add spatial resolution and allow identification and analysis of isolated morphological and functional niches. Analyses of intact tissue sections also circumvent the problem with poor and skewed representation of different thymic cell populations, especially stromal cells, after enzymatic digestion and analysis of solubilized thymic cells (42). Further, the integration of transcriptomic and proteomic data has the potential to overcome limited correlation between mRNA transcripts and protein expression (43, 44, 45). Studies of human 22q11DS thymus are rare since they are constrained by the restricted availability of patient samples, and a key limitation of this study is the small patient cohort. Nonetheless, many of our observations are consistent with, and supported by, what has been demonstrated in mouse models of 22q11DS, thereby strengthening the confidence in our findings.

By applying a high-resolution spatial multiomic approach on thymic samples from 22q11DS patients, we present novel observations regarding major changes in the mesenchymal cell compartment, which may lead to constriction of thymic growth and impair crosstalk between mesenchymal and epithelial cells. We could also confirm previously described changes in cortical and medullary compartments and reduced numbers of thymic Tregs. In addition, we report novel findings of lower total numbers of B cells, higher frequencies of mTEC subsets in the medulla, and an altered inflammatory milieu in a spatial niche in the medulla of potential importance for Treg formation. Although additional mechanistic studies are necessary to confirm causal relationships and underlying mechanisms, the study provides further insight into thymic changes in 22q11DS and is of importance both for basic immunology and for understanding the clinical phenotype in 22q11DS.

## Materials and methods

### Patient samples

Thymus tissue was obtained through a collaboration with the pediatric heart center at the Queen Silvia Children’s Hospital in Gothenburg, Sweden. All samples were obtained from patients who underwent partial thymectomy during open-heart surgery for congenital heart defects. The study comprised 22q11DS patients aged 1–2 mo (*n* = 2, 50% females) and controls aged 3–5 mo (*n* = 8, 50% females) (Table S1). Control samples were collected from patients considered immunologically healthy, with no known immune-related conditions. Tissue was collected directly at surgery in cold medium and further processed within 2 h. Written informed consents were obtained from the patients’ legal guardians. The study was approved by the Regional Ethical Review Board in Gothenburg (dnr 353-92 and dnr 217-12).

### Spatial transcriptomics

Fresh-frozen O.C.T.-embedded tissues were sectioned into capture areas on Visium gene expression slides and further analyzed using Visium Spatial Gene Expression Kit (10X Genomics) according to the manufacturer’s protocol (46). Tissue section thickness was set to 8 µm, and tissue permeabilization time was optimized to 20 min. Indexed libraries were pooled in two groups and sequenced using one lane of Illumina NovaSeq SP-100 and one lane of Illumina NovaSeq 34-300 Reagent Kit with read setup 28-10-10-90. FastQ files were processed using the 10X Space Ranger analysis pipeline, and the generated h5 files were further analyzed in Python using the Scanpy toolkit (47). Quality control was performed jointly for all samples prior to tissue annotation and downstream analysis. Spots with >10% mitochondrial reads and <5% ribosomal genes were filtered out. Genes expressed in <10 spots were removed together with all hemoglobin and mitochondrial genes.

### Tissue annotation and DEG analysis

Annotation of the thymic tissues was performed using the TissueTag Python package following their suggested workflow (25). Cortical and medullary region assignments were initially informed by the expression of selected marker genes, *AIRE* (medulla) and *ARPP21* (cortex), that were spatially mapped onto the hematoxylin- and eosin-stained tissue images. These assignments were subsequently refined by manual annotations of the histological images to delineate pixel-based anatomically refined regions including cortex, medulla, and capsule. The annotations were then mapped to each Visium spot based on spatial proximity, resulting in spot-based annotations of the tissue. CMJ spots were defined using a distance-based criterion, identifying spots closest to the corticomedullary boundary as a transitional region. The spot-based annotations were further validated by the expression of differentially expressed marker genes. This approach resulted in four defined regions: capsular, cortex, CMJ, and medulla that were used for downstream comparative analyses.

### DEG analysis and GO enrichment analysis

Data analysis was performed in Python (version 3.9.21) using Scanpy (version 1.10.2).

Normalization and log transformation using the log(X + 1) formula were performed prior to regression analysis based on the percentage of mitochondrial reads followed by scaling to unit variance and zero mean. DEG between 22q11DS and control thymus, as well as gene expression between each anatomical region, was assessed using the Wilcoxon rank-sum test implemented in scanpy.tl.rank_genes_group. The DEG analyses were performed on raw expression values, and results were considered significant if the adjusted P values (Benjamini–Hochberg) were <0.05. Differentially expressed genes with adjusted P <0.05 and log fold change >0.5 were selected for GO enrichment analysis. GO enrichment was performed using the PANTHER classification system (48) via the GO resource. Enriched GO terms were filtered based on adjusted P value (false discovery rate [FDR]) <0.05 and fold enrichment >2. Highly general GO terms (term size > 600), and non–thymus-relevant and overlapping terms were excluded from further analysis.

### Deconvolution of spatial transcriptomics using scRNA-seq data

Cell-type composition of the Visium data was inferred using publicly available scRNA-sequencing data generated from postnatal thymic tissue (26). Cell annotations and cell numbers used are listed in Table S2. To minimize the impact of low-quality data, spots with <500 expressed genes were removed prior to analysis. Deconvolution was performed using the Cell2location model (27) following the authors’ instructions. Estimation of reference cell-type signatures was performed using a negative binomial regression model accounting for batch effects (donor). The model was trained using 500 epochs. Spatial mapping was computed with N_cell_per_location = 10 and detection_alpha = 20 using 1,000 epochs.

To obtain estimates of cell-type abundance, we used the fifth percentile of the inferred cell abundance distributions. Comparisons of cell-type abundance between 22q11DS and controls were performed using two-sided Mann–Whitney U tests. Spatial co-occurrence of cell types was identified using NMF applied on the estimated cell abundance matrix. The run_colocation package implemented in Cell2location was used using five factors with three restarts. Estimations of cell type–specific gene expression at each spatial location were then assessed using a conditional expected expression framework, as described by Cell2location (initially adapted from the robust cell type decomposition method).

### Spatial proteomics analysis using the CODEX/PhenoCycler platform

Fresh-frozen thymic tissue sections adjacent to the sections used for spatial transcriptomics were used for multiplexed immunofluorescence imaging using the PhenoCycler (formerly CODEX) protocol previously described (23, 24). The 8-µm-thick sections were stained with a 27-marker antibody panel and imaged in a PhenoCycler-Fusion instrument with magnification 20× in 13 different cycles. The antibodies, reporters, and exposure times used are displayed in Table S3. Raw data images of each marker were processed, integrated, and exported as one qptiff file for each sample.

### Complementary immunohistochemical staining

Two antibodies (anti-FOXP3 and anti-AIRE), conjugated in-house, did not yield adequate signal within the CODEX/PhenoCycler workflow and were therefore excluded from the multiplex panel. These markers were instead detected using conventional immunohistochemical staining on separate adjacent tissue sections. 8-µm-thick sections were thawed on Drierite beads before fixation with 4% PFA for 15 min and incubation with blocking buffer (Akoya Biosciences) for 30 min. Sections were incubated overnight at 4°C with primary antibodies, anti-AIRE (cat. no. 14-9534-82; Invitrogen) and anti-FOXP3 (cat. no. 320001; BioLegend), diluted in PBS with 0.3% Triton. The sections were then washed three times with TBS/0.1% Tween-20 and incubated at room temperature for 2 h with secondary antibodies diluted in 0.5% Tris-NaCl blocking buffer (TNB) blocking buffer (Akoya Biosciences) + Hoechst (Invitrogen). After washing three times with TBS/0.1% Tween, the sections were mounted with a coverslip and sealed using Fluoromount-G (Invitrogen). Imaging was performed using a PhenoCycler-Fusion scanner instrument with magnification 20× and exposure time 200 ms. Raw data images were exported as qptiff files for each sample.

### Image processing and cell phenotyping

Initial image processing and cell segmentation were performed using QuPath (version 0.5.1). Single-cell measurements were exported and further analyzed in Python (version 3.9.21) following the analysis pipeline proposed by Akoya Biosciences Academy. Tissue sections exhibiting low signal-to-noise ratio, and nonspecific or oversaturated staining for individual markers were excluded from the analysis of that specific marker. Cell outliers were removed by clipping the value to the mean of top 20 values for each marker. The data were then scaled so that the expression of all markers in all samples was set to a common scale. Scaling was conducted using Scimap’s pp.rescale() function (Gaussian mixture model). Cell phenotyping was performed using Scimap’s tl.phenotype_cells() function with a set gate = 0.75 for CD19, gate = 0.65 for FOXP3, and gate = 0.65 for KRT14. Differences in percentages of CD19^+^ cells and FOXP3^+^ cells in cortex and medulla of 22q11DS and controls were then assessed using sing two-sided Mann–Whitney U tests.

### Immunohistochemical staining of collagens in the thymic tissue

Within 2 h after collection of tissue at surgery, the thymic tissue was cut into small pieces, put into O.C.T. embedding compound, and snap-frozen in isopentane cooled by liquid nitrogen. The O.C.T. blocks were kept in −80°C until analysis. 7-µm sections were put onto SuperFrost glass slides, fixed in cold acetone, and rinsed in PBS before immunostaining. Unspecific epitopes were blocked using blocking buffer (Agilent Dako) with 5% serum from secondary host (goat or donkey) for 15 min. Staining was performed for 1 h at room temperature with primary antibodies (cat. no. MA126771; Thermo Fisher Scientific [anti-collagen I], 14-9871-82 [anti-collagen IV]) diluted in PBS with 0.1% saponin. The sections were washed with PBS before incubation with secondary antibodies + Hoechst diluted in PBS with 0.1% saponin for 30 min at room temperature. After washing with PBS, the sections were mounted using ProLong Gold antifade reagent (Invitrogen) and were left to dry at room temperature overnight in dark. Image acquisition was performed using an Axioscan 7 slide scanning microscope with a ZEISS Colibri 7 camera and a Plan-Apochromat 20×/0.8 M27 objective. Raw image data were exported as qptiff files. Image processing and analysis was performed in QuPath (version 0.5.1).

### Gene expression analysis and copy-number assessment by qPCR

DNA was extracted from frozen O.C.T.-embedded tissue sections using DNeasy Blood and Tissue Kit (cat. no. 69504; Qiagen), while RNA was extracted using RNeasy Micro Kit (cat. no. 74004; Qiagen), according to the manufacturer’s protocol. RNA concentrations and purity were determined using a NanoDrop One spectrometer. cDNA was generated using High-Capacity cDNA Reverse Transcription Kit (cat. no. 4368814; Thermo Fisher Scientific) prior to qPCR assay using 9 μL of diluted cDNA (20 ng/reaction), 10 μL TaqMan Universal PCR Master Mix (cat. no. 4364338; Thermo Fisher Scientific), and 1 μL gene-specific TaqMan probes (Thermo Fisher Scientific, assay IDs: Hs00962558_g [*TBX1*], Hs02511558_s1 [*COMT*], Hs06606866_s1 [*CRKL*], Hs03297761_s1 [*RPPH1*], Hs00164004_m1 [*COL1A1*], Hs00266371_m1 [*COL3A1*], Hs00266237_m1 [*COL4A1*], Hs01095582_m1 [*COL6A1*], Hs02800695_m1 [*HPRT1*], Hs00919266_m1 [*FOXN1*]). The reactions were performed using a QuantStudio 3 PCR instrument. The cycling conditions consisted of an initial incubation at 50°C for 2 min, followed by an initial denaturation at 95°C for 10 min, and then 40 cycles of denaturation at 92°C for 15 s and elongation at 60°C for 1 min, during which fluorescence was measured. All samples were run in triplicates. Ct values of the target genes were normalized to reference genes (*RPPH1* and *HPRT1*). Relative copy number and relative expression (RQ) were determined using the ΔCt method.

### Online supplemental material

The supplemental material includes complementary figures and tables detailing reagent lists, and cell-type definitions used in this study. Table S1 shows patient and control sample characteristics. Table S2 shows cell-type annotations used for Cell2location. Table S3 shows antibodies used for the CODEX protocol. Fig. S1 Annotation of thymic tissue regions and validation by marker gene expression. Fig. S2 Cell-type abundances across thymic regions in controls and 22q11DS. Fig. S3 Alterations in WNT signaling, FOXN1 expression, and spatial niche organization in the 22q11DS thymus.

## Supplementary Material

Table S1 shows patient and control sample characteristics.

Table S2 shows cell-type annotations used for Cell2location.

Table S3 shows antibodies used for the CODEX protocol.

## Data Availability

The raw Visium and PhenoCycler data have been deposited in the Swedish National Data Service (SND, https://snd.gu.se/, a data repository certified by CoreTrustSeal) under the accession code 2026-67 (https://doi.org/10.5878/teyk-ej56) and are available upon reasonable request by completion of a Data Access Request via the SND website.
