# Supplementary material for: Fibroblast-driven collagen expansion and altered thymic medullary niches in 22q11.2 deletion syndrome
Source: J Hum Immun. 2026 May 4;2(4):e20260011. doi: 10.70962/jhi.20260011 (PMC13177773; doi:10.70962/jhi.20260011)
Supplement: Table S1 — shows patient and control sample characteristics. [file jhi_20260011_tables1.docx]

| Healthy controls Sample ID | Sex | Age (months) | Heart defect | Number of Visium spots  after filtering |
| --- | --- | --- | --- | --- |
| 1 | Female | 3.5 | ASD | 4440 |
| 2 | Female | 3 | VSD | 2801 |
| 3 | Male | 3.5 | Tetralogy of Fallot | 3527 |
| 4 | Male | 4 | Tetralogy of Fallot | 3862 |
| 5 | Male | 5 | Pulmonary atresia with VSD | 4358 |
| 6 | Female | 5 | VSD | 4532 |
| 7 | Male | 5 | VSD | 4366 |
| 8 | Female | 5 | VSD | 4417 |
| 22q11DS patients Sample ID |  |  |  |  |
| 1 | Female | 2 | Truncus type 2, VSD, ASD | 3417 |
| 2 | Male | 1 | Tetralogy of Fallot | 1294 |

**Supplemental Table 1. Patient and control sample characteristics**

The table summarizes sample metadata for patients and controls, including sex, age, cardiac diagnosis, and the number of Visium spots included in the analysis for each sample.
