# Supplementary material for: Fibroblast-driven collagen expansion and altered thymic medullary niches in 22q11.2 deletion syndrome
Source: J Hum Immun. 2026 May 4;2(4):e20260011. doi: 10.70962/jhi.20260011 (PMC13177773; doi:10.70962/jhi.20260011)
Supplement: Table S2 — shows cell-type annotations used for Cell2location. [file jhi_20260011_tables2.docx]

**Supplemental Table 2. Cell-type annotations used for Cell2location**

| **Cell type** | **Cell type explained** | **No. of cells** |
| --- | --- | --- |
| DP | Double-positive thymocytes | 44438 |
| DN | Double-negative thymocytes | 8197 |
| CD8+T | CD8 single positive T cells | 7419 |
| CD4+T | CD4 single positive T cells | 7250 |
| αβT(entry) | Early αβ T cells | 6069 |
| Endo | Endothelial cells | 5354 |
| Treg | Regulatory T cells | 5302 |
| mTEC(I) | Medullary thymic epithelial cell, subtype I (*KRT14*+) | 5104 |
| CD8αα | CD8αα⁺ T cells (innate-like T cells) | 4482 |
| B cell | B lymphocytes | 4273 |
| Fb 1 | Fibroblasts, subtype I | 3887 |
| VSMC | Vascular smooth muscle cells | 3022 |
| CD4+Tmem | CD4 positive memory T cells | 2196 |
| T(agonist) | Agonist-selected T cells | 2090 |
| CD8+Tmem | CD8 positive T memory cells | 1643 |
| Fb 2 | Fibroblasts, subtype II | 1523 |
| pDC | Plasmacytoid dendritic cells | 907 |
| γδT | Gamma delta T cells | 538 |
| mcTEC | Bipotent thymic epithelial progenitor cells | 457 |
| mTEC(II) | Medullary epithelial cells, subtype II (*AIRE*-expressing) | 430 |
| NK | Natural killer cells | 379 |
| cTEC | Cortical thymic epithelial cells | 328 |
| DC1 | Conventional dendritic cells type 1 | 312 |
| mTEC(III) | Medullary epithelial cells, subtype III (*KRT1*-expressing) | 258 |
| TEC(neuro) | Neuroendocrine-like thymic epithelial cells | 217 |
| DC2 | Conventional dendritic cells type 2 | 209 |
| aDC | Activated dendritic cells | 200 |
| ILC3 | Group 3 innate lymphoid cells | 160 |
| TEC(myo) | Thymic epithelial cells with myoid-like features | 113 |
| Mono | Monocytes | 104 |
| Mac | Macrophages | 102 |
| ETP | Early thymic progenitors | 20 |
| NKT | Natural killer T cells | 74 |
| Lymph | Lymphatic endothelial cells | 69 |
| mTEC(IV) | Medullary thymic epithelial cells, subtype IV (tuft-like) | 39 |

The table lists annotated cell types from the Park, Botting et al. 2020 human thymus dataset, their corresponding explanations, and the number of cells used for estimation of reference cell type signatures with the Cell2location deconvolution framework.
