# Supplementary material for: Fibroblast-driven collagen expansion and altered thymic medullary niches in 22q11.2 deletion syndrome
Source: J Hum Immun. 2026 May 4;2(4):e20260011. doi: 10.70962/jhi.20260011 (PMC13177773; doi:10.70962/jhi.20260011)
Supplement: Table S3 — shows antibodies used for the CODEX protocol. [file jhi_20260011_tables3.docx]

**Supplemental Table 3. Antibodies used for the CODEX protocol**

| **Marker** | **Clone** | **Akoya** | **Barcode** | **Fluorochrome** | **Exposure** |
| --- | --- | --- | --- | --- | --- |
|  |  |  |  |  |  |
| **CD2** | RPA-2.10 | 4250005 | BX002 | Atto 550 | 200 ms |
| **CD3** | UCHT1 | 4550103 | BX015 | Cy5 | 200 ms |
| **CD4** | SK3 | 4350010 | BX021 | Cy5 | 200 ms |
| **CD8** | SK1 | 4150004 | BX004 | AF750 | 300 ms |
| **CD11c** | S-HCL-3 | 4350012 | BX027 | Cy5 | 250 ms |
| **CD19** | HIB19 | 4350003 | BX003 | Cy5 | 400 ms |
| **CD21** | Bu32 | 4150009 | BX013 | AF488 | 750 ms |
| **CD31** | WM59 | 4250009 | BX032 | Atto 550 | 200 ms |
| **CD34** | 561 | 4250020 | BX035 | Atto 550 | 300 ms |
| **CD38** | FF | 4150007 | BX007 | AF488 | 200 ms |
| **CD45** | HI30 | 4150003 | BX001 | F488 | 200 ms |
| **CD49f** | GoH3 | 4350007 | BX033 | Cy5 | 200 ms |
| **CD69** | FN50 | 4250022 | BX041 | Atto 550 | 300 ms |
| **CD90** | 5E10 | 4150021 | BX022 | AF488 | 300 ms |
| **CD104** | 58XB4 | 4250008 | BX005 | Atto 550 | 300 ms |
| **CD138** | MI15 | 4150008 | BX010 | F488 | 750 ms |
| **CD278 (ICOS)** | C398.4A | 4250013 | BX017 | Atto 550 | 200 ms |
| **CD279 (PD1)** | EH12.2H7 | 42500 | BX014 | Atto 550 | 300 ms |
| **HLA-DR** | L243 | 4250006 | BX026 | Atto 550 | 250 ms |
| **Ki67** | B56 | 4250019 | BX047 | Atto 550 | 250 ms |
| **Podoplanin** | AKYP0007 | 4250094 | BX12 | Atto 550 | 500 ms |
| **CD11b** | ICRF44 | In house | BX031 | Cy5 | 400 ms |
| **Galectin 10** | EPR11197 | In house | BX055 | Cy5 | 400 ms |
| **PDGFR** | EPR220597 | In house | BX030 | Cy5 | 500 ms |
| **CD15** | FUT4 | In house | BX023 | Atto550 | 300 ms |
| **KRT14** | Poly19053 | In house | BX046 | AF647 | 500 ms |
| **Cd1d** | 51.1 | In house | BX006 | AF750 | 300 ms |
